# Flow cytometry analysis of adrenoceptors expression in human adipose-derived mesenchymal stem/stromal cells

**DOI:** 10.1038/sdata.2018.196

**Published:** 2018-10-02

**Authors:** Pyotr A. Tyurin-Kuzmin, Daniyar T. Dyikanov, Julia I. Fadeeva, Veronika Yu. Sysoeva, Natalia I. Kalinina

**Affiliations:** 1 Department of Biochemistry and Molecular Biology, Faculty of Fundamental Medicine, M.V. Lomonosov Moscow State University, Moscow, Russia

**Keywords:** Hormone receptors, Stem-cell research, Mesenchymal stem cells, Flow cytometry

## Abstract

Mesenchymal stem/stromal cells (MSCs) were identified in most tissues of an adult organism. MSCs mediate physiological renewal, as well as regulation of tissue homeostasis, reparation and regeneration. Functions of MSCs are regulated by endocrine and neuronal signals, and noradrenaline is one of the most important MSC regulators. We provided flow cytometry analysis of expression of adrenergic receptors on the surface of human MSCs isolated from ten different donors. We have found that the expression profile of adrenergic receptors in MSCs vary significantly between donors. We also showed that alpha1A-adrenoceptor expression is upregulated under the action of noradrenaline. We share our flow cytometry raw data, as well as processing of these data on a flow cytometry repository for freely downloading.

## Background & Summary

Mesenchymal stem/stromal cells (MSCs) were identified in the stromal-vascular compartment within most adult tissues, including bone marrow, fat and skeletal muscles^1^. MSCs mediate physiological renewal of connective tissues by differentiation into multiple directions, such as bone, fat and cartilage. Another important function of MSCs is paracrine regulation of tissue homeostasis, reparation and regeneration. Functionally, these cells are highly heterogeneous. MSCs functions are under neurohumoral control and one of the most important MSCs regulators is noradrenaline (norepinephrine). Sympathetic neurons were identified as important components of the MSCs niche^2^. Agonists of β-adrenergic receptors inhibit adipogenic differentiation of MSCs into white adipose tissue^3^ and promote differentiation into brown adipocytes^4^. Noradrenaline also inhibits secretory activity of MSCs^2^.

In the study related to this data descriptor^5^, we have shown that β-receptor agonists regulate the combination of receptor isoforms at the cell surface. Thus, the stimulation of MSCs by noradrenaline or dobutamine leads to an increase in expression of α1A-adrenergic receptors. As a result, the number of cells responding to noradrenaline by Ca^2+^-dependent pathways increases up to 2.5 times. At the same time, the expression of β-adrenoceptors at the cell surface decreases^5^. These shifts in receptor expression profiles would result in a switch from the activation of the β-adrenoceptor/cAMP-mediated pathway to the α1A-adrenoceptor/Ca^2+^-mediated one. Such changes in intracellular signalling cascades would therefore lead to distinctive MSC responses.

Here, we shared the flow cytometry raw data, where we analysed the expression of adrenergic receptors at the surface of human MSCs, isolated from 10 different donors. We analysed expression of α1A-, α1B-, α2A-, α2B-, β1-, β2- and β3-adrenergic receptors. We also measured the changes in cell surface expression of adrenoceptors after stimulation of the cells with noradrenaline. The raw data of the flow cytometry experiments and the results of their processing have been uploaded to the open access resource FlowRepository, for downloading. We have found that the expression profile of adrenergic receptors in MSCs varied significantly from donor to donor.

## Methods

### Material collection, MSCs isolation and culturing

MSCs were isolated from abdominal subcutaneous fat tissue harvested during surgical operations from 10 healthy young donors (age 45.25±4.11 years; BMI 23.4±2.5 (TyurinKuzmin_*et al*._Table_1.xlsx, Data Citation 1) using enzymatic digestion. All experiments were carried out in accordance with approved guidelines. All donors gave their informed consent and the local ethics committee of city clinical hospital #31 (Moscow, Russia) approved the study protocol. Donors with infectious or systemic diseases and malignancies were not included in the study. Adipose tissue was washed extensively with 2 volumes of Hank's balanced salt solution (HBSS) with 1% antibiotic/antimycotic solution (HyClone) and then digested at 37 °C for 1 h with equal volumes of collagenase (66.7 U/ml, Sigma-Aldrich) and dispase (10 U/ml, BD Biosciences). Enzyme activity was neutralized by an equal volume of culture medium (AdvanceStem basal medium for human undifferentiated mesenchymal stem cells (HyClone) containing 10% of Advance stem cell growth supplement (CGS) (HyClone), 1% antibiotic/antimycotic solution) and suspension was centrifuged at 200 g for 10 min. Fat droplets and mature adipocytes fell into the top fraction. The fraction of mature adipocytes and supernatant were removed. The cell pellet was resuspended in culture medium and filtered through a 100 μm nylon cell strainer (BD Biosciences). Erythrocytes were removed with lysis buffer. Cells were collected by centrifugation, and resuspended in culture medium. Cells were cultured in AdvanceSTEM Mesenchymal Stem Cell Media containing 10% AdvanceSTEM Supplement (HyClone), 1% antibiotic–antimycotic solution (HyClone) at 37 °C in 5% CO2, in an incubator. Cells were passaged at 70% confluency using HyQTase solution (HyClone). For the experiments, MSCs cultured up to 3rd- 4th passages were used.

### Flow cytometry

MSC immunophenotype and the proportion of cells expressing adrenergic receptors were analysed using flow cytometry. Cells were detached from culture dishes using HyQTase solution and then washed in phosphate buffered saline (PBS - PanEco, Russia) with centrifugation at 300 g for 5 min. Cells were fixed for 15 min with 4% formalin and 1% bovine serum albumin (Sigma, USA) solution in PBS. After fixation, cells were washed twice with PBS (centrifuged at 300 g for 5 min). Cells were stained with antibodies against CD45 (Pacific Blue labelled Mouse anti-Human CD45 antibodies, BioLegend, No 304022), CD73 (R-phycoerythrin (PE) labelled Mouse anti-Human CD73 antibodies, BD Pharmingen, No 550257), CD90 (PE-Cy5, a tandem fluorochrome that combines PE and a cyanine dye, labelled Mouse anti-Human CD90 antibodies, BD Pharmingen, No 555597) and CD105 (fluorescein isothiocyanate (FITC) labelled Mouse anti-Human CD105 antibodies, BD Pharmingen, No 561443) for immunophenotyping the cells. Isotype controls were Pacific Blue labelled Mouse IgG1 (BioLegend, No 400131), PE labelled Mouse IgG1 (BD Pharmingen, No 555749), PE-Cy5 labelled Mouse IgG1 (BD Pharmingen, No 555750), FITC labelled Mouse IgG1 (BD Pharmingen, No 555748). Cells were stained with antibodies against adrenergic receptors α1A (Abcam ab137123, dilution 1:250), α1B (Abcam ab169523, 1:100), α2A (Abcam ab65833, 1:100), α2B (Abcam ab151727, 1:100), β1 (Abcam ab3442, 1:100), β2 (Abcam ab61778, 1:100), β3 (Abnova, H00000155-B01P, 1:100), following by secondary antibodies Alexa Fluor 647 AffiniPure F(ab')_2_ Fragment Donkey Anti-Rabbit IgG (H+L) (Jackson Immunoresearch, 711-606-152, 1:500), Alexa Fluor 594 AffiniPure F(ab')_2_ Fragment Goat Anti-Rabbit IgG (H+L) (Jackson Immunoresearch, 111-586-045, 1:500), Alexa Fluor 488 AffiniPure Donkey Anti-Rabbit IgG (H+L) (Jackson Immunoresearch, 711-545-152, 1:500), DyLight549-conjugate AffiniPure Goat antiMouse IgG (H+L) (Jackson Immunoresearch, 115-505-146) or DyLight649-conjugate AffiniPure F(ab')_2_ Fragment Rabbit Anti-Mouse IgG (H+L) (Jackson Immunoresearch, 315-496-003). Normal rabbit IgG (10500C, Invitrogen, 1:300) and normal mouse IgG1 (Dako X0931, 1:20) were used as a negative control. Stained cells were analysed using the flow cytometry scanner BDLSR Fortessa Special Order Research Product (BD Pharmingen, USA). We measured signal from fluorochromes in the following channels: AlexaFluor 488— excitation 488, emission 530/30, AlexaFluor 594— excitation 561, emission 610/20, AlexaFluor 647 and DyLight649 — excitation 640 emission 670/14, DyLight549 — excitation 561 emission 585/15. To analyse the effect of noradrenaline on adrenergic receptor expression in MSCs, cells were stimulated by noradrenaline for 1 hr, washed three times with HBSS and incubated in full growth medium for 5 hrs followed by flow cytometry assessment. We evaluated the proportion of cells expressing particular adrenergic receptors on 2D flow cytometry plots and histograms using FlowJo V7.6.2 software.

These methods are expanded versions of previously described methods in the study related to this data descriptor^5^.

## Data Records

**Figure 1:**
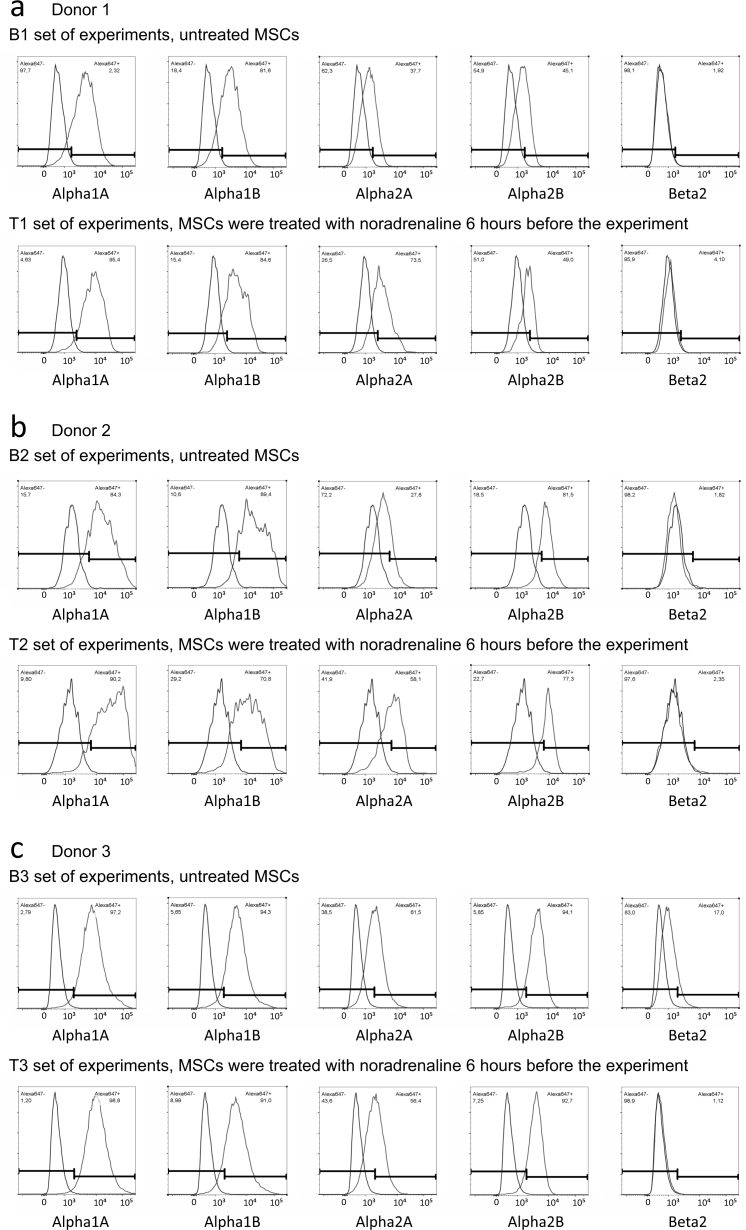
Flow cytometry histograms of experiments shared at FlowRepository. (**a**) Expression of a1A, a1B, a2A, a2B and b2 adrenoceptors in MSCs isolated from Donor 1, B1 and T1 sets of experiments. (**b**) Expression of a1A, a1B, a2A, a2B and b2 adrenoceptors in MSCs isolated from Donor 2, B2 and T2 sets of experiments. (**c**) Expression of a1A, a1B, a2A, a2B and b2 adrenoceptors in MSCs isolated from Donor 3, B3 and T3 sets of experiments. Abbreviations: index “B” means basal level of adrenoceptors in unstimulated MSCs, index “T” means treated cells, MSCs were treated with 10-6 M noradrenaline 6 hours before the experiment.

**Figure 2:**
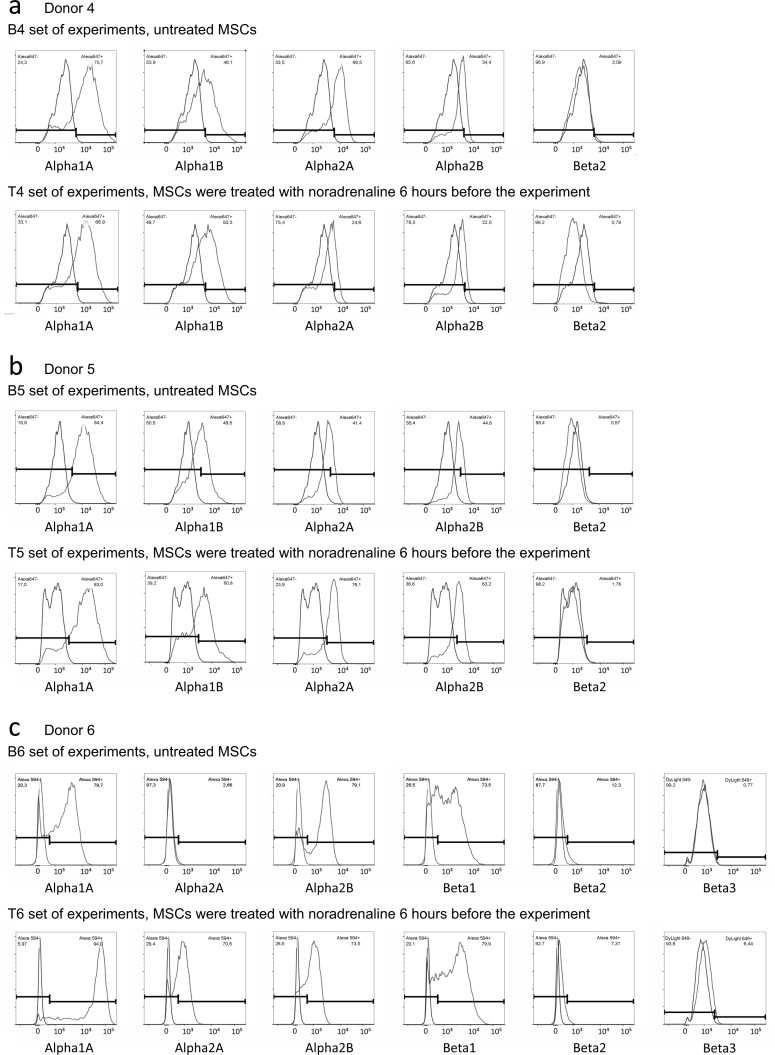
Flow cytometry histograms of experiments shared at FlowRepository. (**a**) Expression of a1A, a1B, a2A, a2B and b2 adrenoceptors in MSCs isolated from Donor 1, B1 and T1 sets of experiments. (**b**) Expression of a1A, a1B, a2A, a2B and b2 adrenoceptors in MSCs isolated from Donor 2, B2 and T2 sets of experiments. (**c**) Expression of a1A, a1B, a2A, a2B and b2 adrenoceptors in MSCs isolated from Donor 3, B3 and T3 sets of experiments. Abbreviations: index “B” means basal level of adrenoceptors in unstimulated MSCs, index “T” means treated cells, MSCs were treated with 10-6 M noradrenaline 6 hours before the experiment.

**Figure 3:**
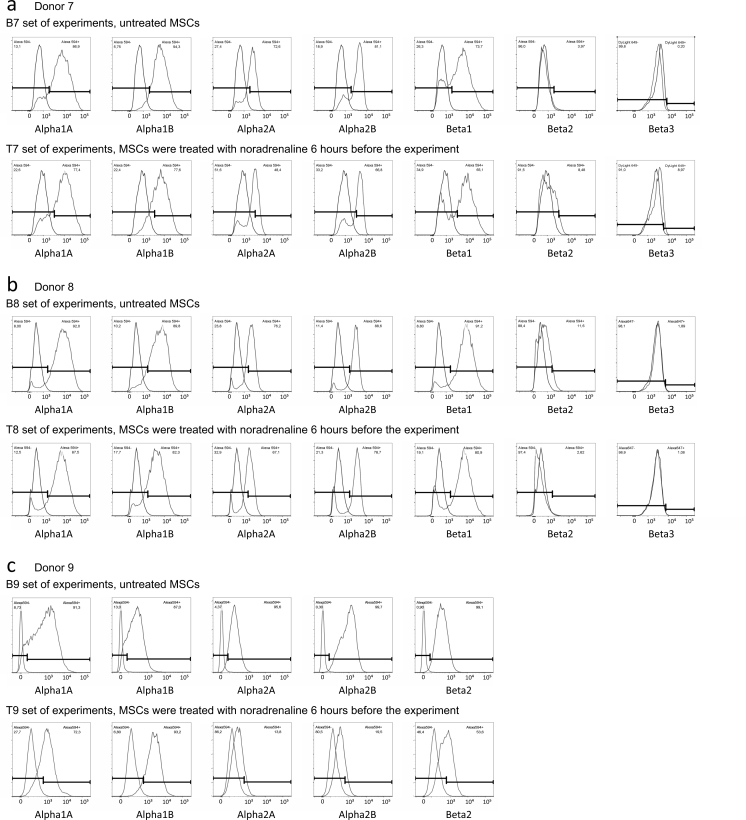
Flow cytometry histograms of experiments shared at FlowRepository. (**a**) Expression of a1A, a1B, a2A, a2B and b2 adrenoceptors in MSCs isolated from Donor 1, B1 and T1 sets of experiments. (**b**) Expression of a1A, a1B, a2A, a2B and b2 adrenoceptors in MSCs isolated from Donor 2, B2 and T2 sets of experiments. (**c**) Expression of a1A, a1B, a2A, a2B and b2 adrenoceptors in MSCs isolated from Donor 3, B3 and T3 sets of experiments. Abbreviations: index “B” means basal level of adrenoceptors in unstimulated MSCs, index “T” means treated cells, MSCs were treated with 10-6 M noradrenaline 6 hours before the experiment.

**Figure 4:**
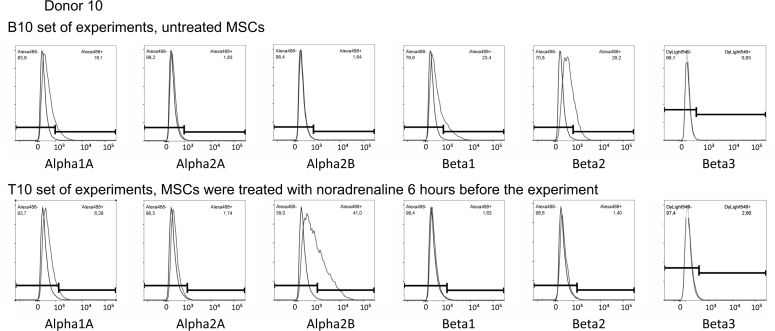
Flow cytometry histograms of experiments shared at FlowRepository. Expression of a1A, a1B, a2A, a2B, b1, b2 and b3 adrenoceptors in MSCs isolated from Donor 10, B10 and T10 sets of experiments. Abbreviations: index “B” means basal level of adrenoceptors in unstimulated MSCs, index “T” means treated cells, MSCs were treated with 10-6 M noradrenaline 6 hours before the experiment.

We have deposited the raw data sets, including two different flow cytometry sets in pair format. Each dataset contains 12-18 samples in two groups, i.e., MSCs pre-treated with noradrenaline versus control cells (these cells were rinsed with growing medium in parallel with noradrenaline treated cells) from the same donor. In every group, expression of α1A, α2A, α2B and β2 adrenergic receptors was analysed (Donors 1-10). α1B adrenergic receptors were analysed in 8 donors (Donors 1-6 and 9-10) and both β1 and β3 adrenoceptors were analysed in 4 donors of 10 (Donors 7-10). Each dataset contains two rabbit IgG controls (one for every group) and, if needed, two mouse IgG controls. Descriptions of raw data files are included in the file TyurinKuzmin_*et al*._Table_2.xlsx (Data Citation 1). Each dataset includes also FlowJo workspace files with processing of appropriate flow cytometry experiments and XML output of the FlowJo workspaces (for descriptions of workspaces and XML files, please see the file TyurinKuzmin_*et al*._Table_3.xlsx (Data Citation 1)). To simplify the data analysis, we also provide flow cytometry histograms for the every experiment (Figures 1, 2, 3 and 4).

The data files and Tables 1-3 were stored in FlowRepository resource (Data Citation 1).

### The reuse value of the data

These data may be useful for the analysis of total expression of adrenoceptors and relative expression of their different isoforms in MSC populations, also for comparable analysis of receptor expression in MSCs from different sources.

## Technical Validation

**Figure 5:**
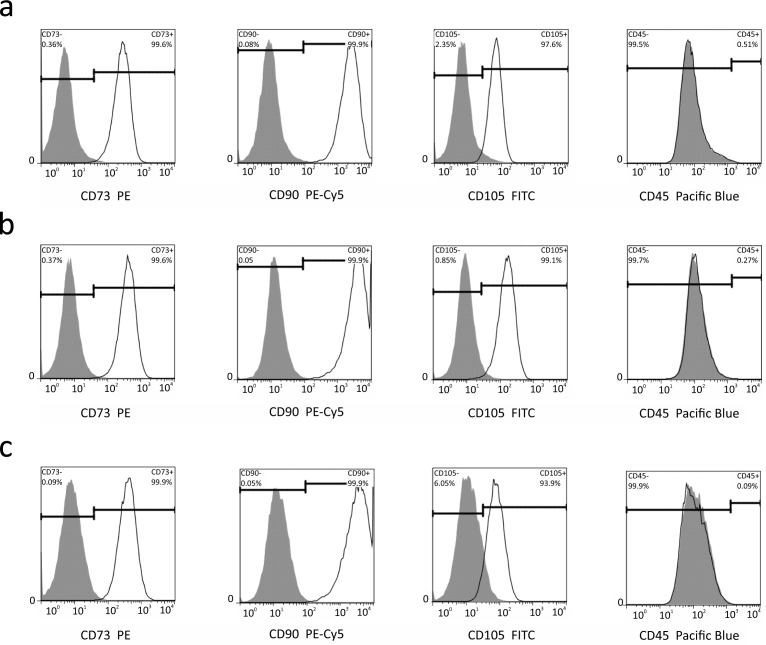
Phenotypic analysis of MSCs isolated from three different donors. Flow cytometry graphs of CD73, CD90 and CD105 surface markers specific for MSCs, and lymphocyte marker CD45. (**a**) Expression of CD73, CD90, CD105 and CD45 in MSCs isolated from Donor 1a. (**b**) Expression of CD73, CD90, CD105 and CD45 in MSCs isolated from Donor 2a. (**c**) Expression of CD73, CD90, CD105 and CD45 in MSCs isolated from Donor 3a. All of the markers were analysed in parallel using four fluorescent channels: PE – R-phycoerythrin (CD73), PE-Cy5 – a tandem fluorochrome that combines PE and a cyanine dye (CD90), FITC - fluorescein isothiocyanate (CD105), and Pacific Blue (CD45). The most of the cells had CD73+ CD90+ CD105+ CD45− phenotype so they were MSCs. Control IgG – grey filled curves, cell surface antigens – black curves. The raw data of these experiments are shared at FlowRepository (Data Citation 1).

The protocol of MSC isolation has been verified in a number of our previous reports where we tested the isolated cells, both on their differentiation capabilities and on the expression of surface markers^5–10^. Here we used a verified protocol, but we additionally tested cells isolated from three different donors for the expression of surface markers specific for MSCs. Isolated cells were 93-100% positive for CD73, CD90 and CD105, and all were negative for CD45. These analyses confirmed that the majority of cells isolated using our protocol were MSCs, as shown in Fig. 5. The raw data of these experiments are shared at the same site as the main experiment on FlowRepository (Data Citation 1).

## Additional information

**How to cite this article**: Tyurin-Kuzmin, P.A. *et al*. Flow cytometry analysis of adrenoceptors expression in human adipose-derived mesenchymal stem/stromal cells. *Sci. Data*. 5:180196 doi: 10.1038/sdata.2018.196 (2018).

**Publisher’s note**: Springer Nature remains neutral with regard to jurisdictional claims in published maps and institutional affiliations.

## Supplementary Material
